# Optimizing Silk Nanoparticle Assembly with Potassium Ions: Effects on Physicochemical Properties and Encapsulation Efficiency

**DOI:** 10.1021/acsabm.5c00598

**Published:** 2025-08-07

**Authors:** Napaporn Roamcharern, Daniel J. Brady, John A. Parkinson, Zahra Rattray, F. Philipp Seib

**Affiliations:** † Strathclyde Institute of Pharmacy and Biomedical Sciences, 3527 University of Strathclyde, 161 Cathedral St., Glasgow G4 0RE, Scotland, U.K.; ‡ Fraunhofer Institute for Molecular Biology and Applied Ecology IME, Branch Bioresources, Ohlebergsweg 12, 35392 Giessen, Germany; § Department of Pure and Applied Chemistry, University of Strathclyde, 295 Cathedral Street, Glasgow G1 1XL, Scotland, U.K.; ∥ 9378 Friedrich Schiller University Jena, Institute of Pharmacy, Department of Pharmaceutical Technology and Biopharmaceutics, Lessingstr. 8, 07743 Jena, Germany

**Keywords:** Bombyx mori, silk fibroin, metal ion, antisolvent precipitation, nanomedicine

## Abstract

Silk fibroin is a promising biomaterial for nanocarrier-based drug delivery due to its biocompatibility, biodegradability, and tunable mechanical properties. In addition, the silk protein is amenable to various processing strategies, offering flexibility for optimizing particle characteristics. Emerging evidence highlights that metal ions can modulate silk conformation and structure in the silk gland, as well as influencing self-assembly, potentially impacting silk nanoparticle fabrication. Our previous study highlighted the potential of Ca^2+^ in silk nanoparticle fabrication. However, other metal ions in the silk gland influence silk fibroin behavior too. Here, we investigate how potassium ions (K^+^), with similar abundance to Ca^2+^ in the silkworm gland, influence silk nanoparticle formation as modulators of self-assembly and material properties, aiming to produce nanoparticles with distinct physicochemical profiles. We show that K^+^ enhances silk assembly, increases nanoparticle size, alters surface charge (zeta potential), and boosts production yield, thereby minimizing silk wastage during silk nanoparticle preparation. Potassium ions also significantly improve payload encapsulation efficiency, making K^+^ inclusion valuable for a range of drug-loading applications. The resulting silk nanoparticles exhibit reduced toxicity and inflammatory response, highlighting their promise as safe and effective nanocarrier candidates for drug delivery. Our findings establish K^+^ as a fundamental yet powerful tool for tuning silk nanoparticle properties to meet pharmaceutical needs.

## Graphical abstract

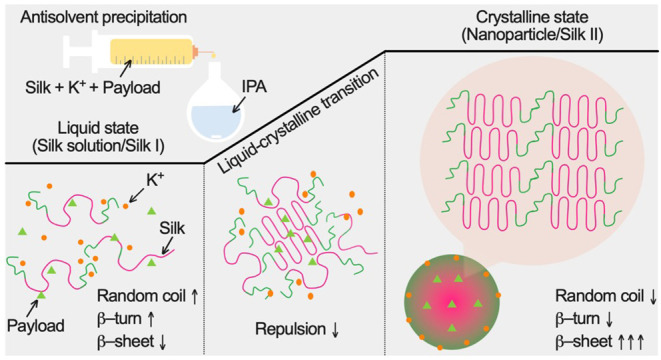

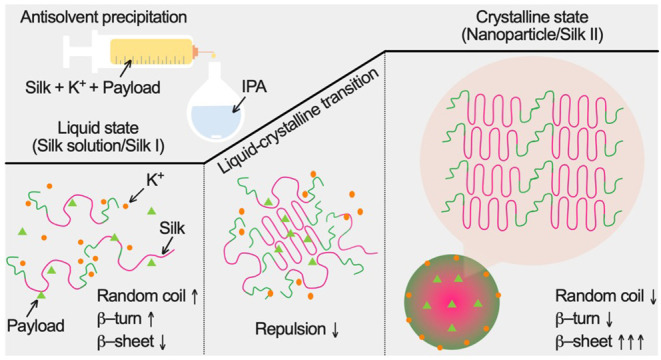

## Introduction

Silk fibroin from the silkworm is recognized as a natural biomaterial with favorable mechanical strength, biocompatibility, and biodegradability^1–4^ and with a broad spectrum of formats available through various processing protocols (reviewed in ref 5,6 ). Silk fibroin-based nanoparticles have been fabricated using regenerated aqueous silk fibroin extracted and dissolved from silk cocoons.^7–9^ Recently, several techniques have been developed for nanoparticle manufacture from liquid fibroin, with antisolvent precipitation confirmed to be a highly effective method with convenient operation, no requirement for expensive apparatus, and desirable efficiency.^10–13^ The antisolvent process involves first dissolving a biopolymer in a solvent in which the material has high solubility, followed by gradual addition of an antisolvent, a liquid in which the biopolymer has low solubility. The antisolvent enhances nucleation, growth, and agglomeration of the biopolymer in the mixed solvent–antisolvent system.^14^

The silk protein structure consists of a light chain (L-chain, ∼26 kDa) linked to the silk heavy chain (H-chain, ∼391 kDa). The heavy chain has a nonrepetitive amino acid sequence located at the C- and N-termini and a unique repeating amino acid sequence of hydrophobic and hydrophilic blocks.^15^ The hydrophobic blocks contain repetitive glycine-X repeats accounting for 94% of H-chain, with X mostly being alanine (A) (65%), serine (S) (23%), or tyrosine (Y) (9%) by composition.^12^ The overall silk H-chain structure is presented as an antiparallel β-sheet structure, stabilized by hydrogen bonds, van der Waals forces, and hydrophobic interactions; therefore, altering the ordered β-sheet degree simply impacts the silk mechanical properties (detailed/reviewed in ref 12,16,17 ).

Liquid silk is affected by various factors, such as pH,^18^ metal ions,^19–21^ shear flows,^22,23^ temperature,^24^ and solvents,^25^ which can influence its conformation. Metal ions are one of the major factors that influence the structure of the liquid silk in the silk gland and play an important role in the spinning of the silk fiber. Our previous study highlighted the significant role played by Ca^2+^ (divalent ion), a key modulator of silk conformation and structure in the silk gland, in the fabrication of silk nanoparticles.^26^ However, changes occur in the conformation and structure of silk during processing due to various factors, notably due to the presence of various metal ions, including K^+^ (monovalent ion), throughout the silk gland.^27^

In nanoparticle formation, the counterion facilitates peptide self-assembly through ionic interactions, with its Debye length influencing whether it preferentially interacts with peptides or water molecules. Ion concentration and solution ionic strength affect the Debye length, thereby regulating self-assembly and particle size via this molecular mechanism, by allowing peptides to come into closer proximity.^28,29^ Divalent and monovalent ions influence the Debye sphere differently, thereby affecting electrostatic interactions in solution. Divalent ions tend to promote more compact assemblies, while monovalent ions result in less impact assemblies.^30^ The aim of the present study was to reveal the effects of K^+^ on silk structure because K^+^ abundance in the silk gland is comparable to that of Ca^2+^ during the storage and spinning processes. In addition, the K^+^ ratio increases significantly from about 3000 μg/g dry weight to 10,000 μg/g dry weight in the gland luminal contents and regulates the silk conformation and stability.^19,31^

Here, we investigated the potential impact of K^+^ on silk processing within our silk nanoparticle manufacturing system that utilizes antisolvent precipitation in a semibatch format. We expected the resulting nanoparticles to display distinct physicochemical properties and favorable characteristics for functioning as nanodrug carriers, particularly after tuning the silk processing protocol for nanoparticle fabrication at the laboratory scale. We also examined the β-sheet-rich structure of silk nanoparticles, as this could drive potential future applications in pharmaceutical design for a variety of hydrophobic drugs and biomolecule loading.^32^ Additionally, we used Thioflavin T as a model hydrophobic drug to examine silk behavior in both liquid form and as a solid nanoparticle, and to assess the impact of K^+^ on silk assembly and encapsulation efficiency. We predict that the findings presented here will provide a development strategy for improving silk-based nanocarrier systems.

## Experimental Section

### Aqueous Silk Preparation

Aqueous silk fibroin was prepared using silk cocoons, following a protocol described elsewhere.^26^ The silk cocoons were chopped into small pieces (5 × 5 mm^2^) after removing contaminants (e.g., soil and dust). A 5 g sample of cocoon pieces was degummed in 2 L of 0.02 M Na_2_CO_3_ by boiling for 1 h under gentle stirring. The degummed silk was washed three times in 1 L of deionized water for 20 min, squeezed to remove excess water, and then dried overnight at ambient temperature in a fume hood.

The dried degummed silk was dissolved in 9.3 M LiBr with a silk:LiBr ratio of 1 g to 4 mL. After incubation at 60 °C for 4 h, the resulting aqueous silk solution was dialyzed against deionized water (Slide-A-Lyzer 3.5K Dialysis Cassette G2, Thermo Scientific, Rockford, IL) for 48 h. The aqueous liquid silk preparation was centrifuged three times at 2885*g* for 40 min at 4 °C (PK 121R Centrifuge, rotor T515, ACL International Srl, Milan, Italy), and the supernatant was transferred to a fresh container and kept at 4 °C until further analysis. The concentration of the aqueous silk solution (% w/v) was calculated as follows.

$$\%\;w/v=\left[\frac{\left(W_{2}-W_{1}\right)}{0.5}\right]\times 100$$

Where, *W*_1_ is the weight of empty weighing boat (g) and *W*_2_ is the weight of the weighing boat containing 0.5 mL silk fibroin after drying at 60 °C for at least 72 h.

### Silk Nanoparticle Manufacture

Silk nanoparticles were synthesized using the antisolvent precipitation method in a semibatch format with previously described optimized parameters.^26^ Potassium ion (K^+^)-induced silk nanoparticles were fabricated at ambient temperature. Briefly, 3% w/v aqueous silk was supplemented with KCl to give mass ratios of K^+^ (mg) to silk (g) of 0, 1.1, and 17.3 mg/g. The highest mass ratio mimicked the K^+^ to silk content in the middle silk gland, while a 2-fold dilution to the lowest ratio (Figure S1) was used to assess concentration-dependent size tuning. The resulting aqueous silk mixtures were added dropwise from a height of 7.5 cm above the meniscus to 30 mL of isopropanol (IPA) using a syringe-pump set at 1 mL/min and a stirring speed of 400 rpm. The silk nanoparticle suspension was centrifuged at 48,400*g* at 4 °C for 2 h, followed by sonication in deionized water. The centrifugation and sonication cycles were repeated twice more, and the final silk nanoparticle product was sonicated and dispersed in deionized water. All silk nanoparticle suspensions were produced in triplicate using different silk batches (*n* = 3).

### Production Yield Determination

The silk nanoparticle production yield was calculated with eq 1.

1 $$\%\;y\mathrm{ield}=\frac{c\mathrm{oncentration}\;\mathrm{of}\;\mathrm{silk}\;\mathrm{nanoparticle}\;\mathrm{suspension}\;\left(\%\;w/w\right)\times m\mathrm{ass}\;\left(g\right)}{c\mathrm{oncentration}\;\mathrm{of}\;\mathrm{aqueous}\;\mathrm{silk}\;\left(\%\;w/v\right)\times v\mathrm{olume}\;\left(\mathrm{mL}\right)}\times 100$$

Where, mass is the total weight of the silk nanoparticle product (g), while concentration of silk solution and volume are initial concentration (3% w/v) and initial volume (6 mL) used in antisolvent precipitation, respectively. The % w/w was calculated as detailed in eq 2

2 $$\%\;w/w=\left[\frac{\left(W_{3}-W_{1}\right)}{\left(W_{2}-W_{1}\right)}\right]\times 100$$

Where, *W*_1_ is the weight of empty weighing boat (mg), *W*_2_ is the weight of weighing boat containing 0.3 mL silk nanoparticle suspension (mg), and *W*_3_ is the weight of weighing boat containing 0.3 mL silk nanoparticle suspension after drying at 60 °C for ≥72 h (mg).

### Thioflavin T Fluorescence Measurement of Aqueous Silk

The K^+^-induced silk fibroin structural changes in solution were studied with a Thioflavin T assay. First, 25 μL of the silk fibroin mixtures (3% w/v silk; 0, 1.1, and 17.3 mg K^+^/ 1 g silk) were incubated with 25 μL of 100 μM Thioflavin T and 50 μL of deionized water. A 60% v/v isopropanol sample served as a β-sheet rich (silk II) control. The mixtures were incubated at ambient temperature for 10 min in the dark, and the bound Thioflavin T was measured (excitation = 440 nm, emission = 475 nm) using a fluorescence microplate reader (Polarstar Omega, BMG Labtech, Ortenberg, Germany).

### Nuclear Magnetic Resonance Spectroscopy

For specific studies, larvae, raised on a 40% artificial diet (CREA, Sericulture Laboratory of Padua, Italy) under controlled conditions of 25 °C ± 1 °C and 75% relative humidity ±5%, were used to produce modified isotope-labeled silk cocoons. In brief, 50 mg of d-glucose-^13^C6 (Cortec, Les Ulis, France) was added to 250 mg of dry artificial diet, and this labeled mixture was fed to the larvae twice daily during days 4–6 of the fifth instar. The larvae were then left to spin their cocoons.

Aqueous silk solution was produced, as detailed above, by adding the modified isotope-labeled silk to unmodified silk at a 1.5:1 protein mass ratio. A 0.5 mL sample of 3% w/v silk containing 0, 1.1, and 17.3 mg K^+^ per 1 g silk was prepared in deionized water. NMR spectroscopy was carried out by a Bruker 600 MHz AVANCE II^+^ NMR spectrometer following a previously reported method.^26^

### Fourier-Transform Infrared Spectroscopy

Fourier-transform infrared (FTIR) spectroscopy was used to investigate the secondary structure content of the liquid silk and nanoparticles. Freeze-dried silk and silk nanoparticles were prepared by freezing at −20 °C overnight, followed by lyophilization at −10 °C and 0.140 mbar for 24 h. Freeze-dried silk and 70% ethanol-treated silk films served as silk I and silk II references, respectively. The FTIR spectra were recorded over a wavenumber range of 400–4000 cm^–1^ with a resolution set at 4 cm^–1^ and scanning times set at 32 and 128 scans for background and sample channel scans, respectively (ATR-equipped TENSOR II FTIR spectrometer, Bruker Optik GmbH, Ettlingen, Germany). The secondary structure was analyzed by deconvoluting the FTIR amide I region (1600–1700 cm^–1^).^26^ Briefly, the wavenumber assignment for the secondary structure component were 1697–1710, 1620–1635, and 1605–1625 cm^–1^ for the total β-sheet structure, 1660–1690 cm^–1^ for the β-turn structure^34^ and 1638–1655 cm^–1^ for the random coil structure. The correlation coefficient (*R*) was calculated using second-derivative amide I spectra in comparison to an air-dried silk film, as outlined in a previous study.^26,33^

### Particle Size Measurement of Silk Nanoparticles

Dynamic light scattering (DLS) was used to determine particle size, while nanoparticle tracking analysis (NTA) was employed to measure both particle size and concentration. DLS was carried out on 1:25 (v/v) ratio mixtures of 1 mg/mL silk nanoparticle suspension in deionized water. Analysis was performed under the following conditions: a count rate of 203.6 kcps, a measurement time of 70 s, a measurement position of 3 mm, and a temperature set at 25 °C (Zetasizer Nano-ZS, Malvern Instruments, Worcestershire, U.K.).

Particle size and particle concentration (particle/mL) were studied with NTA (NTA 3.4 Build 3.4.003, Malvern Panalytical Ltd., Worcestershire, U.K.) at 25 °C. Briefly, the silk nanoparticle suspension was diluted in sterile-filtered Milli-Q water to achieve a particle count of 20–80 particles per frame. The setup included an sCMOS camera, a 488 nm laser, a syringe pump set to a speed of 100 (Harvard Apparatus Model 98–4730 Syringe Pump, Massachusetts), and a viscosity value of 1.0000 cP.

NTA data were recorded over five replicate 60 s videos using the light-scattering mode. The capture settings were optimized for silk nanoparticle characteristics, with a screen gain of 3 and camera level of 9 for smaller particle samples (0 and 1.1 mg K^+^), and a screen gain of 1 and camera level of 8 for larger particle samples (17.3 mg K^+^). The data were analyzed using NTA 3.4 (version 3.4.003 software), with a detection threshold set at 6. Each silk nanoparticle type was analyzed in triplicate, with five technical replicates per replicate.^35^

### Zeta Potential Measurement of Silk Nanoparticles

Zeta potential was measured using electrophoretic light scattering (ELS). Briefly, 5 mg/mL of silk nanoparticle suspension was diluted to a ratio of 1:20 (v/v) nanoparticles to deionized water using the following settings: temperature, 25 °C; count rate, 61.5 kcps; zeta run, 12; and measurement position, 2 mm (Zetasizer Nano-ZS, Malvern Instruments, Worcestershire, U.K.).

### Analysis of Silk Nanoparticle Morphology

Silk nanoparticle suspensions were prepared at a concentration of 1 mg/mL in deionized water. A 20 μL aliquot of the suspension was applied to a 5 × 5 mm^2^ silicon wafer (Ted Pella, Inc., CA), left to dry at ambient temperature for 24 h, and gold-coated by sputtering for 40 s from a 35 mm height with a pressure of 0.08 mb and current of 30 mA (Agar Scientific Manual Sputter Coater, Agar Scientific Ltd., Essex, U.K.). Silk nanoparticles were then observed using field emission scanning electron microscopy (FE-SEM) with a 5 kV voltage at magnifications of 10,000×, 20,000×, and 60,000× (Hitachi SU6600, Hitachi High-Tech Europe GmbH, Krefeld, Germany). Circularity and size of silk nanoparticles were analyzed using ImageJ (NIH National Institute of Mental Health, Bethesda, MD) (Figure S2).

### Model Drug Encapsulation in Silk Nanoparticles

Thioflavin T was used as a model drug for loading studies. To prepare Thioflavin T-loaded silk nanoparticles, aqueous silk mixture was supplemented with 14.3 μM Thioflavin T, following silk nanoparticle manufacture. The resulting silk nanoparticles were collected and analyzed using NTA in light scattering mode, followed by acquisition in fluorescent mode with a minor modification for the focus setting. The proportion of empty particles was determined by subtracting the size distribution profile measured in fluorescence mode—which detects Thioflavin T-loaded particles—from that measured in light scattering mode, which detects all particles. The Thioflavin T loading efficiency was calculated as follows

3 $$\mathrm{loading}\;\mathrm{efficiency}\;\left(\%\right)=\frac{\mathrm{particle}\;\mathrm{concentration}\;\mathrm{of}\;\mathrm{silk}\;\mathrm{nanoparticles}\;\left(\mathrm{fluorescence}\;\mathrm{mode}\right)}{\mathrm{particle}\;\mathrm{concentration}\;\mathrm{of}\;\mathrm{silk}\;\mathrm{nanoparticles}\;\left(\mathrm{scattering}\;\mathrm{mode}\right)}\times 100$$

### Cytotoxicity of Silk Nanoparticles

The cytotoxicity of silk nanoparticles was investigated in RAW 264.7 murine macrophages (ATCC, product number TIB-71TM, ATCC, U.K.). Briefly, cells were cultured in Dulbecco’s Modified Eagle Medium (DMEM) supplemented with 10% v/v FBS, 50 U/mL penicillin, and 50 mg/mL streptomycin. When the cells reached 80–90% cell confluency, they were scraped from plates, centrifuged at 380*g* for 4 min, seeded into a 96-well plate at a cell density of 15,000 cells/cm^2^, and incubated in a 5% v/v CO_2_ incubator at 37 °C for 24 h. The culture medium was then replaced with fresh medium containing 31–500 μg/mL of silk nanoparticles, and the cells were cultured for 48 h. Cell viability was determined by adding 20 μL of 5 mg/mL 3-[4,5-dimethylthiazol-2-yl]-2,5 diphenyl tetrazolium bromide solution, incubating for 4 h, and reading the absorbance at 570 nm (Multiskan Ascent V1.24, Thermo Fisher Scientific Inc., MA). Cells treated with blank complete medium served as untreated control cells. Percentage viability was expressed relative to the untreated control, and a cytotoxicity assay was performed with three biological replicates (*n* = 3).

### Inflammation Induction by Silk Nanoparticles

RAW 264.7 murine macrophages were processed as described earlier. After plating and a 24 h incubation, cells were exposed to fresh medium containing 500 μg/mL silk nanoparticles. Cells treated with 200 ng/mL lipopolysaccharide (LPS) served as positive inflammatory-activated control cells. After a 24 h incubation, the medium was collected and centrifuged at 21,630*g* for 5 min (VWR Micro Star 30 centrifuge, VWR International, LLC., Leicestershire, U.K.). The resulting supernatant was transferred to a low-binding protein microtube and preserved at −80 °C for further analyses.

Endogenous nitrite (NO_2_^–^) was measured using a Total Nitric Oxide and Nitrate/Nitrite Parameter Assay Kit (R&D Systems, Minneapolis, MN). Tumor necrosis factor α (TNF-α) was measured using a Mouse TNF-α Quantikine ELISA Kit (R&D Systems, Minneapolis, MN). Cytokine and chemokine profiles were semiquantitatively measured using the Proteome Profiler Array (Mouse Cytokine Array Panel A, R&D Systems, Minneapolis, MN).

### Analysis Software and Statistical Analyses

Data were compiled using Microsoft Excel version 16.92 (Microsoft Office 365 for Mac, Redmond, WA). FTIR data were processed using Origin 2019b (OriginLab, Northampton, MA), and the NMR spectra were analyzed and plotted using MestReNova (Mestrelab Research, Santiago de Compostela, Spain). ImageJ (NIH National Institute of Mental Health, Bethesda, MD) was used to derive size and circularity parameters from scanning electron microscopy micrographs and for cytokine dot intensities on cytokine membranes. Graphs and statistical analyses were prepared using GraphPad Prism 10 (GraphPad Software, Boston, MA). Statistical tests included one-way ANOVA followed by Dunnett’s multiple comparisons test. Asterisks indicate statistical significance, and the number of experimental repeats (*n*) is provided in the figure legends.

## Results and Discussion

### Silk Structural and Conformational Changes

This study explored the role of K^+^ in silk nanoparticle assembly deploying physicochemical solution and solid-state techniques. The Thioflavin T assay was used to assess the impact of K^+^ on liquid silk structural and conformational changes (but also as a model drug as detailed later). Thioflavin T is a fluorescent dye widely used for the determination of protein physicochemical properties, such as the degree of aggregation/hydrophobicity, structural and conformational alterations, and protein interactions (reviewed in ref 36). The intercalation of the Thioflavin T molecule within the β-sheet of the amyloids creates a strong fluorescence signal with an excitation at 440 nm and an emission at 480 nm.^37^ The observed increase in fluorescence intensity (emission wavelength of 475 nm) in the isopropanol-treated aqueous SF condition indicated the bound Thioflavin T state and served as a positive control for the silk β-sheet structure and/or the induction of silk aggregation. However, the use of 1.1 mg or 17.3 mg K^+^ did not significantly alter the silk structure or aggregation compared to the control (0 mg K^+^), suggesting that the Thioflavin T assay was not able to uncover subtle differences in liquid silk interactions and structural property determinations, as modulated by K^+^ treatment in the 1.1–17.3 mg K^+^ range (Figure 1a). The impact of calcium ions on the liquid silk structure, which was also studied with the Thioflavin T assay, was discussed in our previous work.^26^

**Figure 1:**
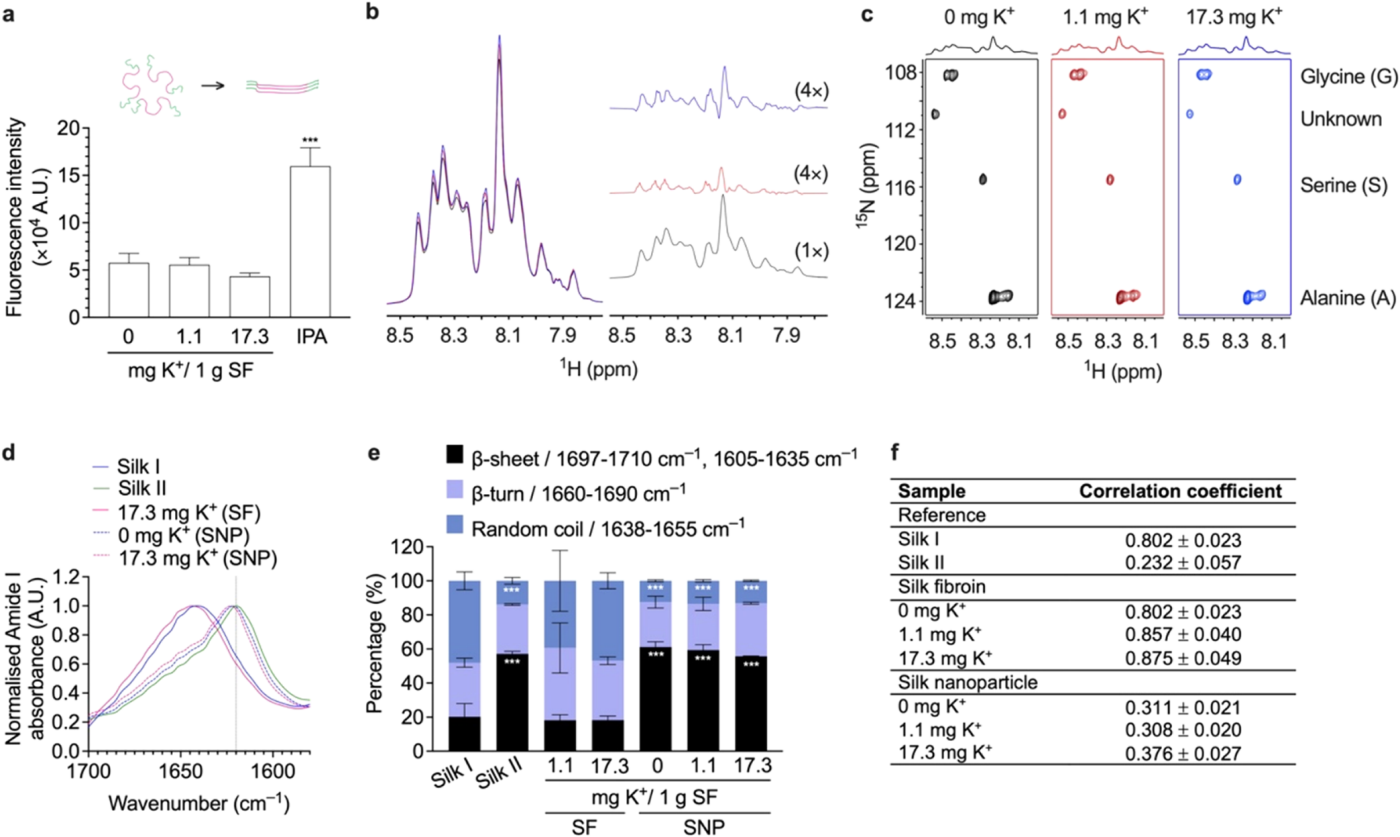
Analysis of the structure and conformation of aqueous silk and solid nanoparticles. (a) The effect of K^+^ on aqueous silk fibroin (SF) structural alteration, as studied using Thioflavin T assays (ThT). The IPA-treated SF indicated the silk β-sheet structure and aggregation control, showing increased fluorescence intensity of bound ThT state (*n* = 3), (b) 1D ^1^H NMR (*n* = 1), (c) and 2D [^1^H, ^15^N] HSQC NMR spectra in the NH region (*n* = 1). The 1D 1H-NMR spectra are shown on the left side of the panel, with the difference spectra (red and blue) displayed on the right for comparison to the control spectrum (black). Black traces represent the control, red corresponds to 1.1 mg K^+^ (4.1% increase), and blue to 17.3 mg K^+^ (5.2% increase). The different spectra were adjusted by multiplying the vertical scale by a factor of 4. (d) The secondary structures of freeze-dried aqueous silk (17.3 mg K^+^) and silk nanoparticles (SNP) (0 and 17.3 mg K^+^) were studied with FTIR and compared to freeze-dried aqueous silk (0 mg K^+^) with low (silk I) and EtOH-treated air-dried silk film with high (silk II) β-sheet content; normalized amide I absorbance spectra, (e) secondary structure content, (f) and correlation coefficient (*n* = 3). The second-derivative amide I spectrum of an air-dried silk film (silk I) served as a reference for the correlation coefficient (*R*) calculations (mean ± SD, *n* = 3). The controls employed in all experiments were derived from the data set previously reported,^26^ carried out at the same time as this study. Statistical analyses: One-way ANOVA and Dunnett’s multiple comparisons test for the Thioflavin T assay; Two-way ANOVA and Dunnett’s multiple comparisons test for secondary structure content; *p* < 0.05 (*), *p* < 0.01 (**), and *p* < 0.001 (***). Abbreviations: ANOVA: analysis of variance; IPA: isopropanol; SF: aqueous silk fibroin; SNP: solid silk nanoparticle; ThT: Thioflavin T; EtOH: ethanol

We also used NMR to explore silk behavior in solution but observed no significant chemical shifts in the NH region (8.5–7.8 ppm) of 1D ^1^H NMR spectra. However, a similar trend to that seen previously with Ca^2+^ was observed, as the K^+^ mass ratio increased, resulting in an increased peak intensity and altered peak sharpness. Compared to the control (0.0 mg K^+^), the spectra for silk formed in the presence of 1.1 mg and 17.3 mg K^+^ displayed overall increases of 4.1 and 5.2%, respectively (Figure 1b). We hypothesize that this increased signal reflected alterations in the exchange rate of labile NH protons. If we assume that the increased signal is linked to a slower exchange rate, this suggests that the NH protons remain attached to nitrogen for a longer period. This increase in the NMR signal suggests that a larger proportion of the labile hydrogens are located in the same local NH chemical environment, implying a change in protein stability.^38^

The 2D [^1^H, ^15^N] HSQC NMR spectra displayed ^15^N chemical shifts for amino acids in a random coil state, corresponding to glycine (108.8 ppm), serine (115.7 ppm), alanine (123.8 ppm), and an unidentified amino acid (110.8 ppm) (Figure 1b).^39^ The amino acids revealed by the 2D [^1^H, ^15^N] HSQC NMR spectra included glycine, alanine, and serine as the major components of silk (Figure 1c), as reported elsewhere,^40^ and accounted for 45.6, 29.8, and 10.7% by mol, respectively. Thus, a notably high intensity was noted for the 2D [^1^H, ^15^N] HSQC NMR spectra contour plot. The hydrophobic amino acids, consisting of glycine-X repeats (review in ref 12,41 ), play an important part in the antiparallel β-sheet structure of silk, as their properties govern the overall characteristic of antiparallel β-sheet structure. However, the regulation relevant to the antiparallel β-sheet structure could be identified according to the main roles of these amino acids: glycine maintains proper packing/assembly,^42^ alanine maintains stability and rigidity,^43^ and serine provides hydrophobicity.^43^ Taken together, the findings suggest that an increase in the proportion of labile hydrogens may predominantly occur in the NH microenvironment of these major amino acids to affect the formation as well as the rigidity of the β-sheet structures, leading to increased aggregation/assembly of the silk fragments.

The FTIR analysis was performed to track changes in the secondary structure of silk from liquid silk to silk nanoparticles prepared using K^+^ at low and high mass ratios (1.1 and 17.3 mg). In the absence of K^+^, the silk nanoparticles exhibited a higher degree of β-sheet structure than the K^+^ silks; however, all silk nanoparticles displayed a typical shift in the IR absorbance spectrum at ∼1640 to ∼1620 cm^–1^ (the amide I region) associated with the β-sheet structure^44^ (Figure 1d). This result was consistent with an increase in the total β-sheet content, from 20.21 to 61.16%, for silk nanoparticles (Figure 1e). This suggests that our nanoparticles exhibited a β-sheet-rich structure that was nearly identical to a silk II reference (a silk film treated with 10% ethanol), consisting of 61% β-sheet, 26% β-turn, and 13% random coil. Interestingly, the incorporation of 1.1 and 17.3 mg K^+^ in silk nanoparticles and liquid silk had no significant impact on the secondary structure content, as the nanoparticles and liquid silk structures were maintained close to those of their silk II and silk I references, respectively (Figures 1e and S3). The correlation coefficient (R) for the nanoparticles, obtained from the second derivative amide I spectra and indicating formulation-induced structural changes in the silk nanoparticles relative to air-dried silk films (silk I reference), ranged between 0.308 and 0.376, similar to the silk II reference value of 0.232 (Figure 1f).

Our FTIR findings suggest that K^+^ mass ratios ranging from 1.1 to 17.3 mg (equivalent to 1 to 17 mM KCl) may not be sufficient to directly influence the structural rearrangement of liquid silk. This was consistent with a previous report showing that higher KCl concentrations (>0.1 M KCl) increased the β-sheet content in spider silk.^45^ One possible explanation is that silk–ion binding interactions in our system may be moderated and saturated in liquid silk, which reaches the maximum β-sheet structure for silk nanoparticles (55.71–61.16%). However, our NMR data prompted further investigation of the effects of K^+^ on silk conformation and assembly, particularly with respect to the modulation of nucleation, saturation, and particle growth during antisolvent precipitation.

### Silk Nanoparticle Physicochemical Properties

Silk nanoparticles were fabricated using low and high K^+^ mass ratios (1.1 and 17.3 mg), and the physicochemical properties, particularly the particle size and polydispersity index (PDI) were assessed using DLS and NTA. Particle size measurements from DLS were consistent with those from NTA (in brackets), showing a slight increase in particle size with higher K^+^ mass ratios compared to 0 mg K^+^ control silk nanoparticles, from 92.98 d·nm (78.81 d·nm) to 105.3 d·nm (80.71 d·nm) for 1.1 mg K^+^, and 290.6 d·nm (171.9 d·nm) for 17.3 mg K^+^. All silk nanoparticles had a favorable PDI of <0.15.^46,47^ However, a significant difference in size between the DLS and NTA determinations was observed for the 17.3 mg K^+^ nanoparticles, which showed a reduction in particle size of ∼ 1.7-fold when determined using NTA, while the control particles and 1.1 mg K^+^ particles exhibited size reductions of ∼1.2-fold and 1.3-fold, respectively. Despite the larger particle size, the 17.3 mg K^+^ silk nanoparticles showed a lower particle concentration compared to the other ones (Figure 2a). Moreover, the 2-fold increase in the K^+^ mass ratio—from the lowest at 1.1 mg to the highest at 17.3 mg—was also investigated, revealing a ratio-dependent effect of K^+^ on size tuning and increased production yield, while maintaining PDI value (<0.15) and negligible reduction of zeta potential (Figure S1). These findings demonstrate that our K^+^ mass ratios enable the tuning of silk nanoparticle size, with the hydrodynamic diameter increasing as the K^+^ mass ratio rises. This result may be attributed to the influence of K^+^ on the physicochemical properties of silk, suggesting that an optimal K^+^ mass of less than 4.3 mg is favorable for achieving particle diameters typical of drug nanocarriers (<200 nm), prolonging a circulation half-life: eliminated by kidneys (<10 nm) and promoted clearance pathway (>200 nm).^48,49^ Although larger in size, 200–300 nm particles may be useful for nanoparticle-mediated topical drug delivery.^50^ This observation was consistent with the FE-SEM micrographs, which confirmed an increase in the particle size with an increase in the K^+^ mass ratio from 63.56 d·nm to 66.17 d·nm for 1.1 mg K^+^ silk nanoparticles and 163.70 d·nm for 17.3 mg K^+^ silk nanoparticles. Interestingly, K^+^ had a negligible effect on particle morphology, as the computed circularity value was maintained close to 1 (ranging from 0.82 to 0.87), similar to that of a perfect sphere^51^ (Figure 2b).

**Figure 2:**
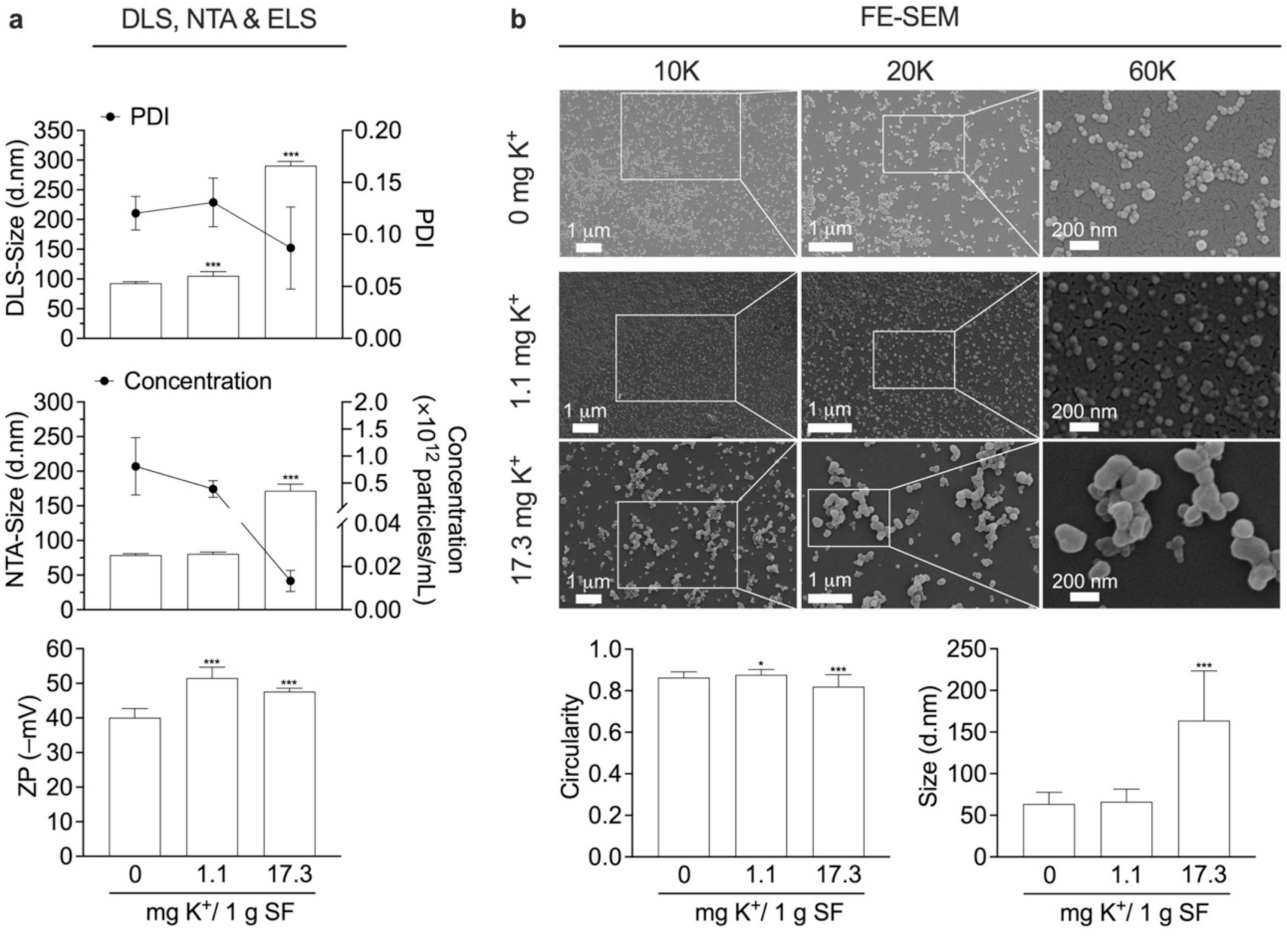
Silk nanoparticle physicochemical properties. (a) Analysis of particle size (using DLS and NTA) and zeta potential (ZP) using ELS (*n* = 3). (b) Analysis of particle size and morphology (using FE-SEM). The FE-SEM images were taken at 10, 20, and 60K magnifications. Size and circularity calculations were performed using 400–500 particles and 100–150 particles, respectively, derived from at least three different regions of interest. The controls employed in all experiments were derived from a previously reported data set,^26^ carried out at the same time as this study. One-way ANOVA and Dunnett’s multiple comparison test were used for statistical analysis: *p* < 0.05 (*), *p* < 0.01 (**), and *p* < 0.001 (***). Abbreviations: ANOVA: analysis of variance; DLS: dynamic light scattering; ELS: electrostatic light scattering; FE-SEM: field emission scanning electron microscopy; NTA: nanoparticle tracking analysis; PDI: polydispersity index; SF: aqueous silk fibroin; ZP: zeta potential.

One possible explanation for the observed effects of K^+^ could involve nucleation, the initial phase in which small clusters of protein fragments begin to aggregate. As the system reaches saturation, the concentration of protein fragments becomes maximal and limits further aggregation (detailed/reviewed in ref 14,33,52 ). As the K^+^ concentration increases, these positive ions shield the negative charges of the silk fragments, reducing electrostatic repulsion between the silk molecules and disrupting the hydration layers around silk molecules. This allows the silk molecules to come closer together, facilitating self-assembly,^28,29,53,54^ and altering the solution’s ionic strength,^55^ which in turn affects protein folding and promotes increased silk assembly. During the particle growth phase, the initially formed aggregates may be more strongly influenced by K^+^ to promote larger clusters from the start. Thus, they continue to increase in size as additional silk fragments join, and this growth is further influenced by the presence of K^+^ and isopropanol. This indicates that size tuning of the nanoparticles is governed by K^+^ in a concentration-dependent manner.

In addition to the particle size, the particle net charge surface was significantly altered by K^+^, as indicated by the increase in zeta potential (ZP) from −40 mV to −52 mV for the 1.1 mg K^+^ silk nanoparticles and to −48 mV for the 17.3 mg K^+^ particles (Figure 2a). The lower negative zeta potential value may reflect a modification of the net charge surface on the particles by the counterions. We speculate that the added KCl (K^+^/Cl^–^) interacted with negatively charged amino acid residues located predominately at the hydrophilic spacer and N-terminus of the silk H-chain,^56^ causing its exposure on the particle surface and altering the net charge surface and particle–solvent interactions (diffusivity and the solvation shield).

### Model Drug-Loaded Silk Nanoparticle

The model drug-loaded silk nanoparticles were fabricated to assess their potential therapeutic efficiency as a drug delivery system. We investigated the interaction between our silk carrier and Thioflavin T, used as a model drug, with a focus on the payload loading efficiency influenced by K^+^. Part of our goal was to explore a molecule designed for multiple functions, including the detection of β-sheet structures and providing fluorescence for easy detection using nanoparticle tracking analysis in the fluorescence and light scattering modes.

The light scattering mode detects all particles based on their light scattering properties, whereas the fluorescence mode detects only those particles exhibiting fluorescence—in this case, due to entrapped Thioflavin T—allowing for the monitoring and tracking of Thioflavin T-loaded particles within the crude silk nanoparticle suspension. The overlaid size distribution data from fluorescence and light scattering modes allows for the calculation of Thioflavin T entrapment efficiency by comparing particle concentrations.^57^ Thioflavin T-loaded nanoparticles were detected using NTA, revealing an increase in the Thioflavin T-loading percentage from 76.24% for the control silk nanoparticles (0 mg K^+^) to 78.27% for the 1.1 K^+^ silk nanoparticles and to 80.10% for the 17.3 mg K^+^ silk nanoparticles (Figure 3a). For the same protein concentration (1 mg/mL), increasing K^+^ mass ratios increased the mean particle size from 100.27 ± 1.36 for the control particles to 95.67 ± 2.89 for the 1.1 K^+^ particles and to 190 ± 2.11 d·nm for the 17.3 mg K^+^ particles. In contrast, fewer particles were detected after 60 s, consistent with the increased K^+^ mass ratios from 7.96 × 10^13^ particles for the control to 13.5 × 10^13^ for the 1.1 K^+^ nanoparticles and 1.9 × 10^13^ particles for the 17.3 K^+^ nanoparticles (Figure 3b).

**Figure 3:**
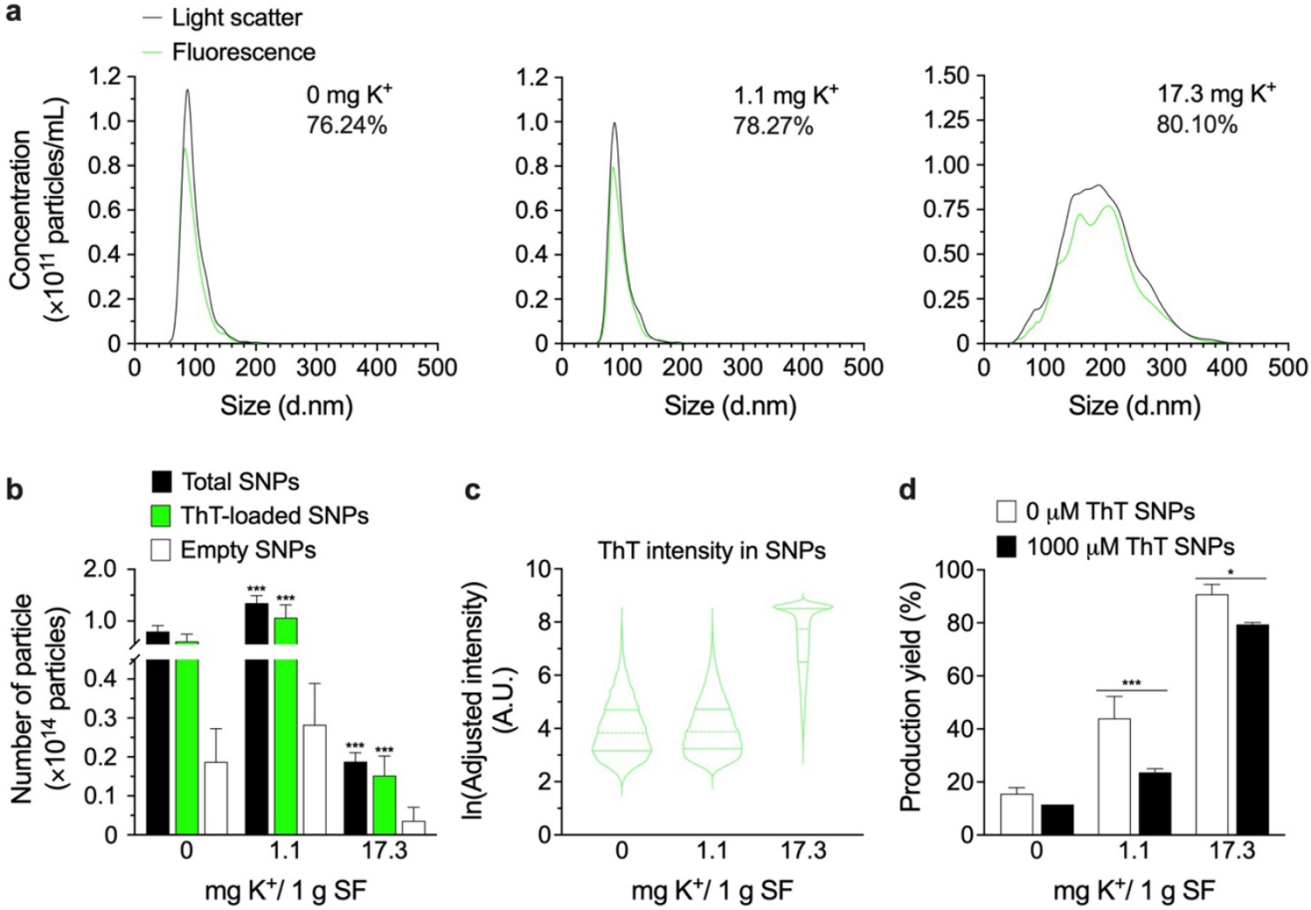
Model drug-loaded silk nanoparticles (SNPs) using Thioflavin T as a drug model. Thioflavin T-loaded SNPs were prepared at 1 mg/mL based on protein concentration and were characterized by NTA (*n* = 3): (a) size distribution profile measured by light scattering mode (black) and fluorescence mode (green); (b) the particle number; (c) Thioflavin T fluorescence intensity. (d) The production yield was calculated according to the SNP dried weight. The controls employed in all experiments were derived from the data set previously reported,^26^ carried out at the same time as this study. One-way ANOVA and Dunnett’s multiple comparison tests were used for statistical analysis: *p* < 0.05 (*), *p* < 0.01 (**), and *p* < 0.001 (***). Abbreviations: ANOVA: analysis of variance; NTA: nanoparticle tracking analysis; SF: aqueous silk fibroin; SNP: silk nanoparticle; ThT: Thioflavin T.

The fluorescence intensity detected from Thioflavin T-loaded silk nanoparticles indicated that Thioflavin T was more efficiently entrapped in larger particles fabricated with a high K^+^ mass ratio (7.4 A.U.), whereas the median intensity showed no significant changes with the low K^+^ mass ratio silk nanoparticles (4.1 A.U.) compared to the 0 K^+^ nanoparticles (4.0 A.U.) (Figure 3c). Comparison of the production yield of Thioflavin T-loaded particles to particles fabricated without Thioflavin T also revealed that the addition of Thioflavin T reduced silk nanoparticle production yield by factors of 1.35, 1.86, and 1.14 for the control, 1.1 mg K^+^, and 17.3 mg K^+^ particles, respectively. However, we suggest that our manufacturing conditions can significantly reduce silk biomaterial wastage, potentially increasing the yield by 2.8-fold (for 1.1 mg K^+^ silk nanoparticles) and 5.8-fold (for 17.3 mg K^+^ silk nanoparticles) compared to control silk nanoparticles during empty particle production (without payloads). Similarly, the yield for Thioflavin T-loaded particle production was 2.1-fold and 6.9-fold greater than for control silk nanoparticles (Figure 3d).

Taken together, our findings suggest that K^+^ elevates the intercalation of the Thioflavin T molecule within the silk assembly/aggregates, as Thioflavin T preferentially interacts with the hydrophobic regions of the protein (e.g., its aggregates and β-sheet structures) (reviewed in ref 58), leading to increased Thioflavin T entrapment in particles with larger sizes. This result can explain why larger particles contain a higher proportion of Thioflavin T and therefore show a higher fluorescence intensity. Compared to biodegradable nanoparticles exemplified by poly­(lactic-*co*-glycolic) acid (PLGA), which offers high encapsulation efficiency for hydrophilic drugs,^59^ or chitosan, known for its versatile applications,^60^ silk nanoparticles offer unique advantages due to the ability to control their particle size^61^ and release profiles,^62^ making them ideal for drug delivery. Beyond confirming the core β-sheet–rich structure of the resulting K^+^-mixed silk nanoparticles (with a size tuning ability ranging from ∼100 to 200 nm), as determined by FTIR, our exploration of the effectiveness of model drug encapsulation has highlighted the benefits of the β-sheet-rich properties of these silk nanoparticles for potential future applications in pharmaceutical dosage form design for a spectrum of hydrophobic drugs and biomolecules.^32^

### Cytotoxicity and Inflammation of Silk Nanoparticles

The cytotoxicity of silk nanoparticles was investigated in macrophages within the 31–500 μg/mL concentration range. All silk nanoparticles showed similar cytotoxicities, as indicated by reductions in cell viability from 99% to 75% in parallel with increased silk nanoparticles concentrations (Figure 4a). Silk nanoparticles at the highest nanoparticle dose (500 μg/mL) triggered negligible release of cytokines and chemokines from macrophages and the response was similar to baseline. In contrast, cells treated with 200 ng/mL LPS (positive control inflammation-stimulated cells) exhibited an excessive release of inflammation-related cytokines and chemokines. Notably, changes in expression levels due to silk nanoparticle treatment were observed only for RANTES, MIP-2, TNF-α, SDF-1, MIP-1β, MIP-2, JE, IP-10, IL-1ra, IFN-γ, and ICAM-1. The expression of other inflammatory markers (e.g., TREM-1, TIMP-1, IL-27, IL-10, IL-6, IL-1α, GM-CSF, and G-CSF) was not elevated in silk-treated cells (Figures 4b and S4). Overall, exposure to silk nanoparticles resulted in negligible cytokine and chemokine release, with expression levels remaining near baseline. No significant increases in NO_2_^–^ (1.2 vs 1.3 μM) or TNF−α (0.02 vs 0.06 ng/mL) were observed when comparing cells before vs after treatment with silk nanoparticles at the highest dose of 500 μg/mL. Conversely, treatment with 200 ng/mL LPS promoted an excessive level of both NO_2_^–^ (4.9 μM) and TNF-α (5.02 ng/mL) (Figure 4c).

**Figure 4:**
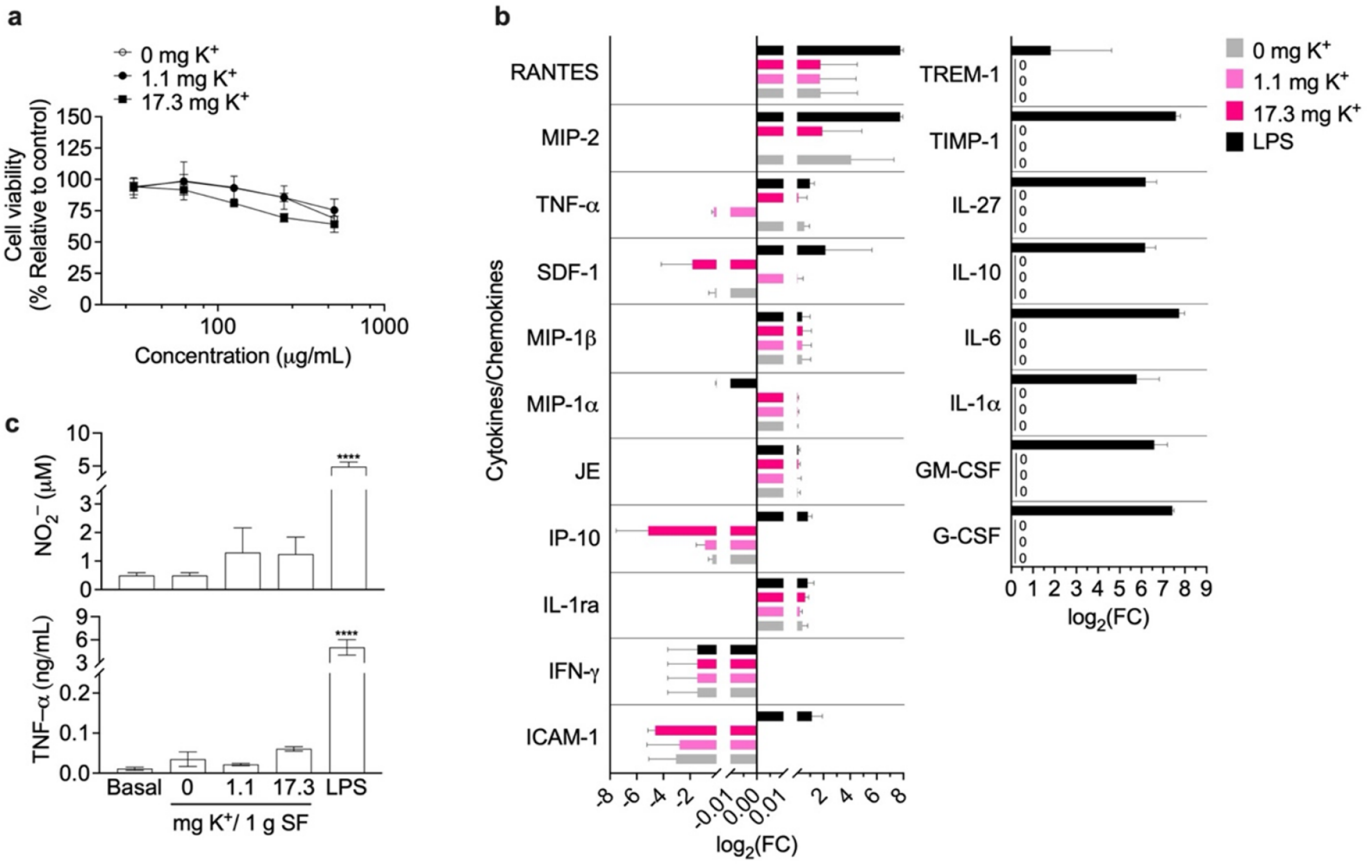
Cellular responses of RAW 264.7 murine macrophages to silk nanoparticles (SNPs). (a) Cytotoxicity of macrophages exposed to SNP levels ranging from 31.25 to 500 μg/mL for 48 h (*n* = 3). Inflammatory responses: (b) release profile of cytokines and chemokines presented in log_2_ fold-change (FC) of expression level compared with a basal control. Interpretation of log_2_(FC): a value of zero indicates no change in expression, positive values indicate upregulation, and negative values indicate downregulation (*n* = 3); and (c) NO_2_^–^ and TNF-α production in macrophages exposed to 500 μg/mL SNPs for 24 h (*n* = 3). Cells treated with 200 ng/mL LPS served as the positive inflammation-stimulated control. Cells exposed only to the complete medium served as a basal control. The controls employed in all experiments were derived from the data set previously reported,^26^ carried out at the same time as this study. One-way ANOVA and Dunnett’s multiple comparison test were used for statistical analysis: *p* < 0.05 (*), *p* < 0.01 (**), and *p* < 0.001 (***). Abbreviations: ANOVA: analysis of variance; LPS: lipopolysaccharide; SF: aqueous silk fibroin; SNP: silk nanoparticle.

Our results showed the high biocompatibility of silk nanoparticles toward macrophages, with no significant induction of inflammatory responses. We propose that the electrostatic interaction network formed by the counterion^28,29,53,54^ contributes to the stabilization of the β-sheet-rich structure. In combination with the negative surface charge, this enhances particle integrity and colloidal stability, promotes shape and size retention, reduces agglomeration, and ultimately lowers cytotoxicity, as destabilized particles may pose a greater risk to normal cell function.^63^ Furthermore, particle size and surface charge may play important roles in cellular responses. With a comparable zeta potential across all samples, smaller particles possess a higher charge density, which can lead to varying degrees of particle–cell interactions, potentially disrupting membrane integrity and triggering programmed cell death pathways (reviewed in ref 64). However, a slight decrease in cell viability was observed with largest (17.3 mg K^+^) silk nanoparticles, suggesting that charge density was of limited biological importance. Overall, these findings demonstrate that the inclusion of K^+^ in our manufacturing protocol generated silk nanoparticles that exhibited high biocompatibility with macrophages, negligible cytotoxicity, and minimal induction of inflammation. Therefore, we propose that K^+^ inclusion serves as a practical additive for tuning silk nanoparticles.

### Impact of Ions on the Modulation of Silk Nanoparticle Properties

Metal elements can influence silk fibroin behavior in the natural spinning environment by regulating its structural transition, stability, and assembly.^31^ We propose a mechanism by which selected ions contribute to the modulation of silk nanoparticles fabrication in vitro using the antisolvent precipitation method. In a previous study, we investigated the effect of Ca^2+^ on silk nanoparticles within the same manufacturing system and established a fine-tuning process model with optimized physicochemical properties. In the present study, we focus on the role of K^+^, which is among the most abundant of the metal elements. Unlike Ca^2+^, K^+^ differs in both valency and its concentration gradient along the silk gland. While Ca^2+^ decreases from the posterior to the anterior region, K^+^ shows a significant increase in the anterior part.^31^ We analyzed the correlation for each key variable factor between K^+^ particles and reference Ca^2+^ particles using correlation coefficients (Figure 5). This result demonstrated that K^+^ and Ca^2+^ both modulated particle size, shape, and loading efficiency in a consistent and correlated manner, suggesting complementary mechanisms. Nonetheless, Ca^2+^ had a greater impact on modulating silk fibroin behavior, promoting more assembly and aggregation and improving production yield by reducing silk fibroin wastage.^26^ We hypothesized that in the aqueous silk state, Ca^2+^ has a greater impact than K^+^ in altering aqueous silk fibroin behavior, as evidenced by Thioflavin T assay and NMR analysis, which showed a difference of up to 17.5% for Ca^2+^ compared to 7% for K^+^. This could be attributed to differences in valency and ionic radius, with Ca^2+^ being more effective than K^+^ at forming strong electrostatic interactions with proteins.^30,53,54,65,66^ However, K^+^ had a much lower impact on disrupting the silk nanoparticle surface charge, whereas Ca^2+^ reduced the ZP by approximately half. Together, our findings show that mimicking the natural roles of K^+^ (this study) and Ca^2+^ (previous study)^26^ improves the physicochemical properties and scalability of silk nanoparticles. These distinct effects highlight the potential of exploring synergistic or antagonistic interactions among multiple metal ions in silk nanoparticle formulation to achieve more tailored properties.

**Figure 5:**
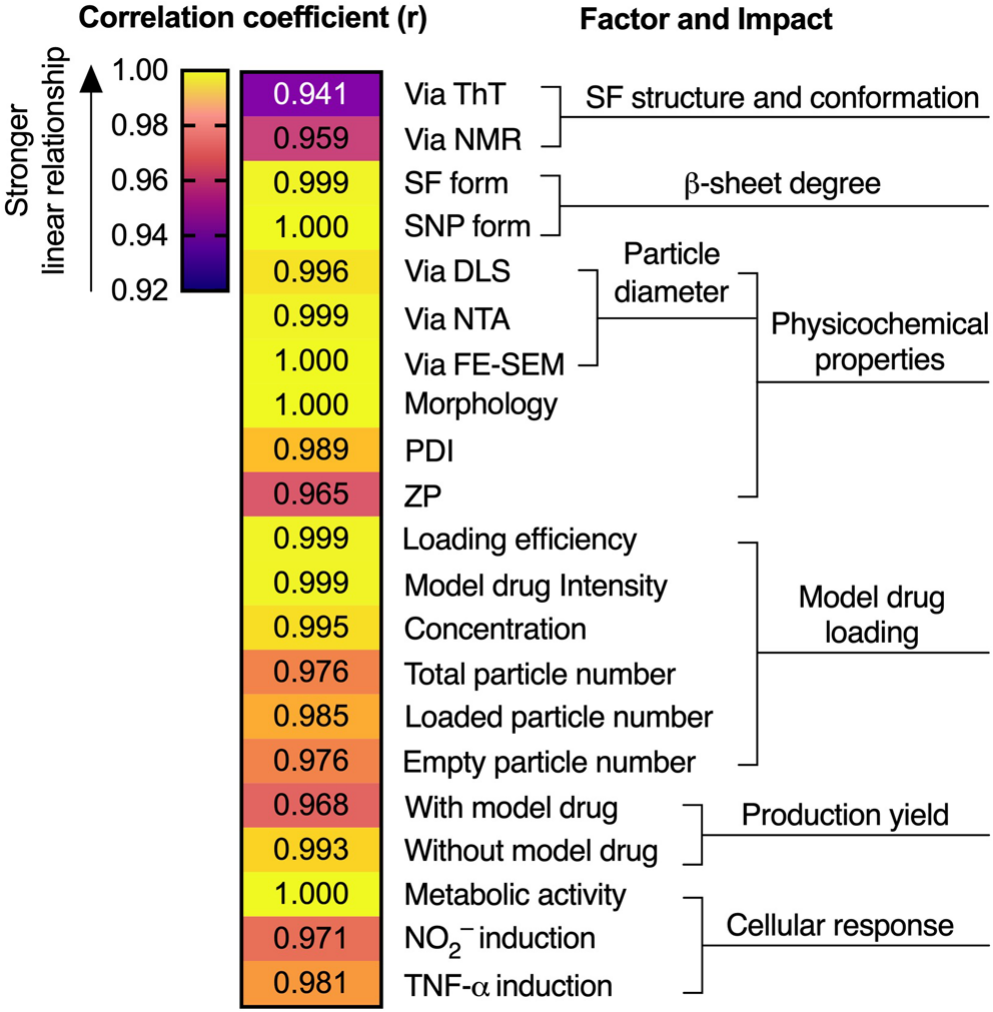
Impact of calcium and potassium on silk nanoparticle performance. The correlation coefficient (r) between the measured variables in the K^+^ data set (this study) and the reference Ca^2+^ data set (previous study^26^) (*n* = 3): positive linear correlation (*r* > 0), nonlinear correlation (*r* = 0), and negative linear correlation (*r* < 0). Abbreviations: SF: aqueous silk fibroin; SNP: silk nanoparticle; ThT: Thioflavin T; NMR: Nuclear magnetic resonance; DLS: Dynamic light scattering; NTA: Nanoparticle tracking analysis; FE-SEM: Field-emission scanning electron microscopy; PDI: Polydispersity index; ZP: zeta potential.

## Conclusions

We manufactured silk nanoparticles using the antisolvent precipitation method in a semibatch format to orchestrate nanoparticle assembly. Our findings demonstrated that K^+^ can modulate the physicochemical properties of silk, both in liquid form and as solid silk nanoparticles. Specifically, K^+^ induced changes in silk self-assembly, promoted the formation of β-sheet-rich cores, enabled tunable particle size, enhanced payload encapsulation efficiency, and favorable shifts in particle net surface charge (zeta potential). The addition of K^+^ also improved formulation efficiency by increasing yield and reducing silk loss during production. The resulting β-sheet-rich silk nanoparticles exhibited low toxicity and minimal inflammatory activity, supporting their potential as biocompatible drug nanocarriers. Collectively, these findings offer a practical strategy for fine-tuning silk-based nanomaterials for pharmaceutical applications. Future work should assess loading capacity for diverse drug types, optimize semibatch scale-up, and evaluate nanoparticle stability under varied storage conditions to support clinical and commercial translation.

## Supplementary Material



## Data Availability

All data created during this research are openly available from the University of Strathclyde-Pure, at DOI: 10.15129/e025fd4c-4651-45b8-8611-6546dced6528.

## Notes

Silk nanoparticle physicochemical properties (Figure S1); schematic of silk nanoparticle circularity and size assessment using ImageJ software (Figure S2); the exemplary IR fitting of the amide I region was performed for K^+^-mixed silk fibroin (1.1 and 17.3 mg K^+^ SF) and K^+^-mixed silk nanoparticles (SNP) (0, 1.1, and 17.3 mg K^+^ SNP), and compared with freeze-dried silk powder (silk I) and EtOH-treated silk film (silk II) controls (Figure S3); inflammatory cytokine levels were measured in RAW 264.7 murine macrophages in response to silk nanoparticle treatment (Figure S4) (PDF)

## Abbreviations

DLS: dynamic light scattering
DMEM: Dulbecco’s modified eagle medium
EtOH: ethanol
FTIR: Fourier-transform infrared
G-CSF: granulocyte colony stimulating factor
GM-CSF: granulocyte-macrophage colony-stimulating factor
HSQC: heteronuclear single quantum correlation
IL-1α: interleukin-1α
IL-1ra: interleukin-1 receptor antagonist
IL-6: interleukin-6
IL-10: interleukin-10
IP-10: interferon γ-induced protein 10
CCL2: JE
IPA: isopropanol
MCP-1: monocyte chemoattractant protein-1
MIP-1α: macrophage inflammatory protein-1α
MIP-1β: macrophage inflammatory protein-1β
MIP-2: macrophage inflammatory protein-2
NMR: nuclear magnetic resonance
NO_2_^–^: nitrite
PDI: polydispersity index
K^+^: potassium ion
RANTES: regulated upon activation, normal t cell expressed and presumably secreted
SF: silk fibroin
SNP: silk nanoparticle
ThT: thioflavin T
TIMP-1: tissue inhibitor matrix metalloproteinase 1
TNF-α: tumor necrosis factor α
TREM-1: triggering receptor expressed on myeloid cell 1
ZP: zeta potential
