# Supplementary material for: Optimizing Silk Nanoparticle Assembly with Potassium Ions: Effects on Physicochemical Properties and Encapsulation Efficiency
Source: ACS Appl Bio Mater. 2025 Aug 7;8(8):6854–64. doi: 10.1021/acsabm.5c00598 (PMC12370172; doi:10.1021/acsabm.5c00598)
Supplement: Supplementary file 1 [file mt5c00598_si_001.pdf]

Supporting Information

Optimizing Silk Nanoparticle Assembly with Potassium Ions: Effects on Physicochemical Properties and Encapsulation Efficiency

Napaporn Roamcharern[a], Daniel J. Brady[b], John A. Parkinson[c], Zahra Rattray[a], F. Philipp Seib[a,b,d*]*

[a]Strathclyde Institute of Pharmacy and Biomedical Sciences, University of Strathclyde, 161 Cathedral St., Glasgow G4 0RE, Scotland, UK

[b]Fraunhofer Institute for Molecular Biology and Applied Ecology, Branch Bioresources, Ohlebergsweg 12, 35392 Giessen, Germany

[c]Department of Pure and Applied Chemistry, University of Strathclyde, 295 Cathedral Street, Glasgow G1 1XL, Scotland, UK

[d]Friedrich Schiller University Jena, Institute of Pharmacy, Department of Pharmaceutical Technology and Biopharmaceutics, Lessingstr. 8, 07743 Jena, Germany

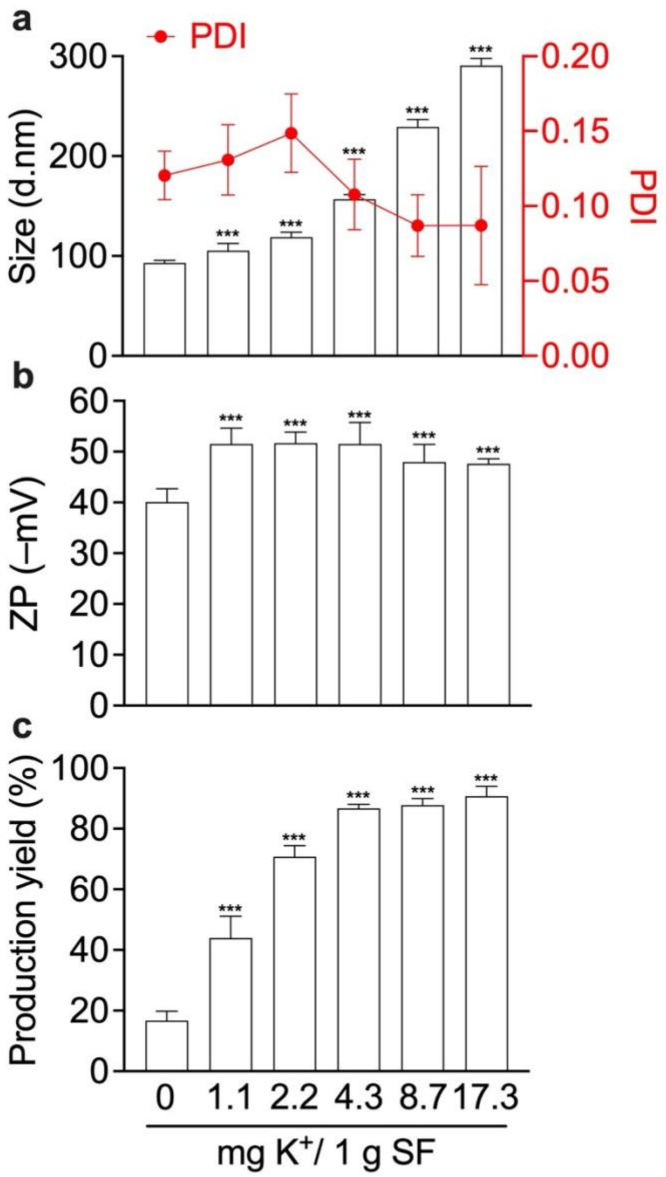

Figure S1. Silk nanoparticle physicochemical properties. **(a)** Analysis of particle size using DLS (**b**) zeta potential (ZP) using ELS, and (**c**) production yield calculated by SNP dried weight ($n = 3$). Silk nanoparticle was manufactured in the K^+ mass range of 1.1 to 17.3 mg. The One-way ANOVA and Dunnett's multiple comparison test were used for statistical analysis: $p < 0.05$ (*), $p < 0.01$ (**), and $p < 0.001$ (***). Abbreviations: ANOVA: analysis of variance; DLS: dynamic light scattering; ELS: electrostatic light scattering; PDI: polydispersity index; SF: aqueous silk fibroin; ZP: zeta potential.

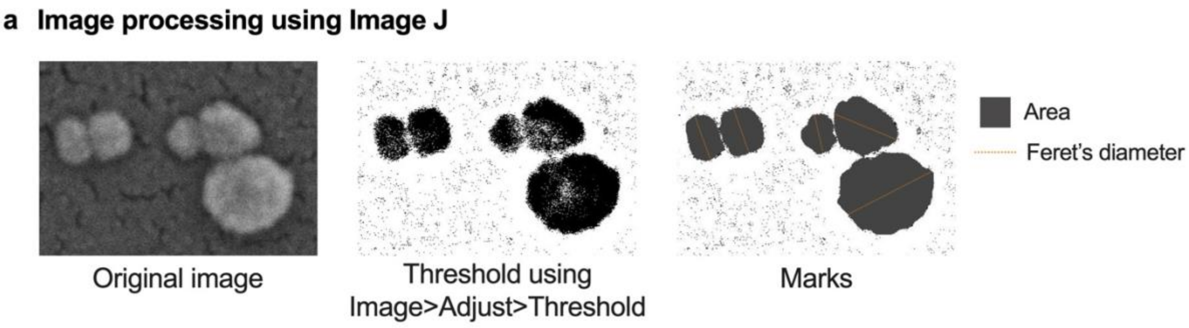

b Analyze > Set measurement > Measure

Interested parameters	Equation/Definition	Interpretation
Circularity	$\frac{4\pi \times \text{area}}{\text{Perimeter}^2}$	1.0 = Perfect circle<br>0.0 = Elongated shape
Feret's diameter	The longest distance between any two points along the selection boundary.	Longer = Larger particle size<br>Shorter = Smaller particle size

Figure S2. Schematic of silk nanoparticle circularity and size assessment using ImageJ software. (**a**) Image processing: threshold setting and particle boundary selection. (**b**) Circularity and Feret's diameter are analyzed by the function of Analyze, Set measurement, and Measure.

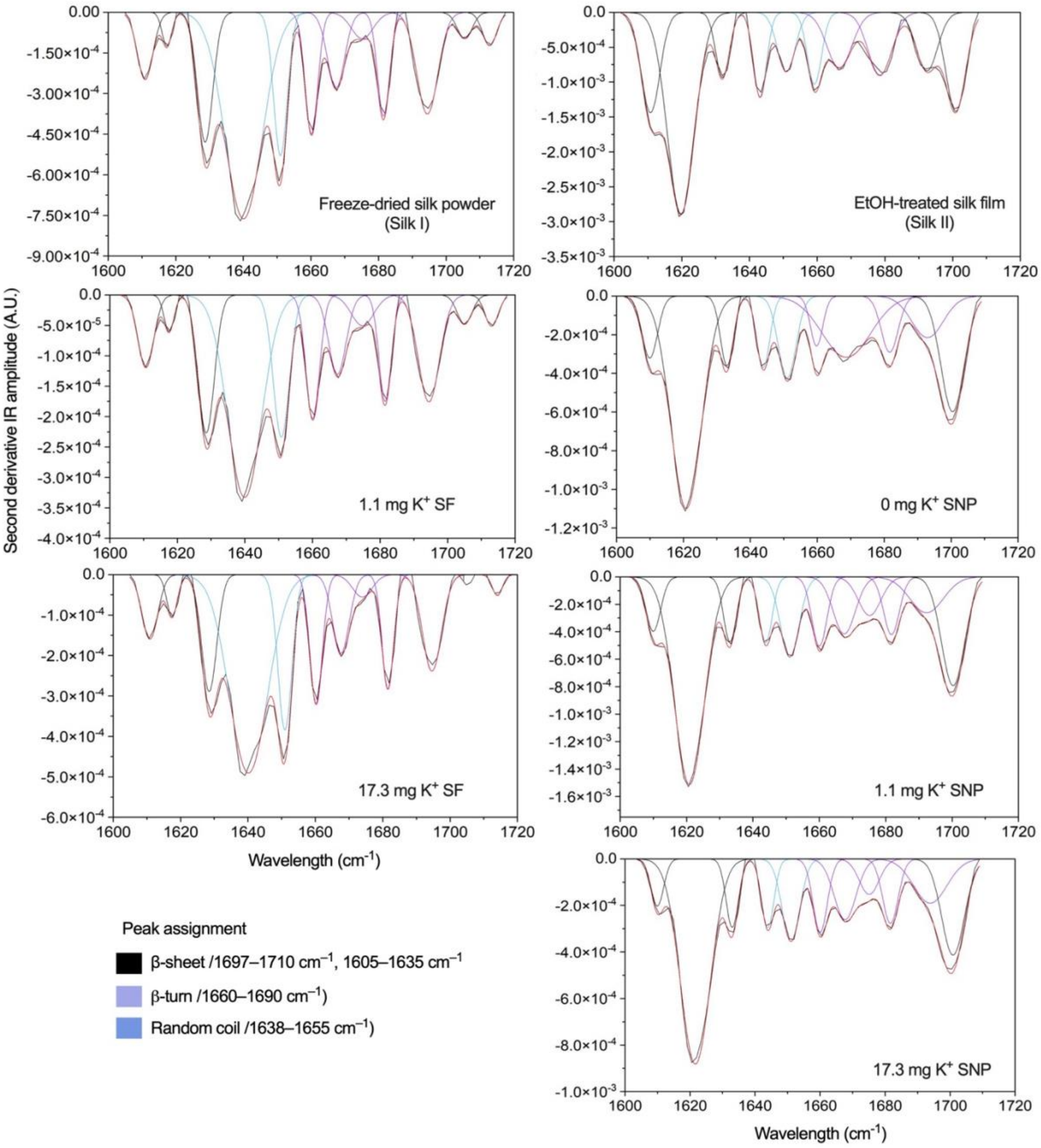

Figure S3. The exemplary IR fitting of the amide I region was performed for K^+-mixed silk fibroin (1.1 and 17.3 mg K^+ SF) and K^+-mixed silk nanoparticles (SNP) (0, 1.1, and 17.3 mg K^+ SNP), in comparison with freeze-dried silk powder (silk I) and EtOH-treated silk film (silk II) controls. The freeze-dried silk powder (silk I) serves as the 0 mg K^+-mixed silk (n = 1). This experiment was carried out alongside our calcium silk nanoparticle,[26] with the same set of controls used for both. Abbreviations: EtOH: Ethanol.

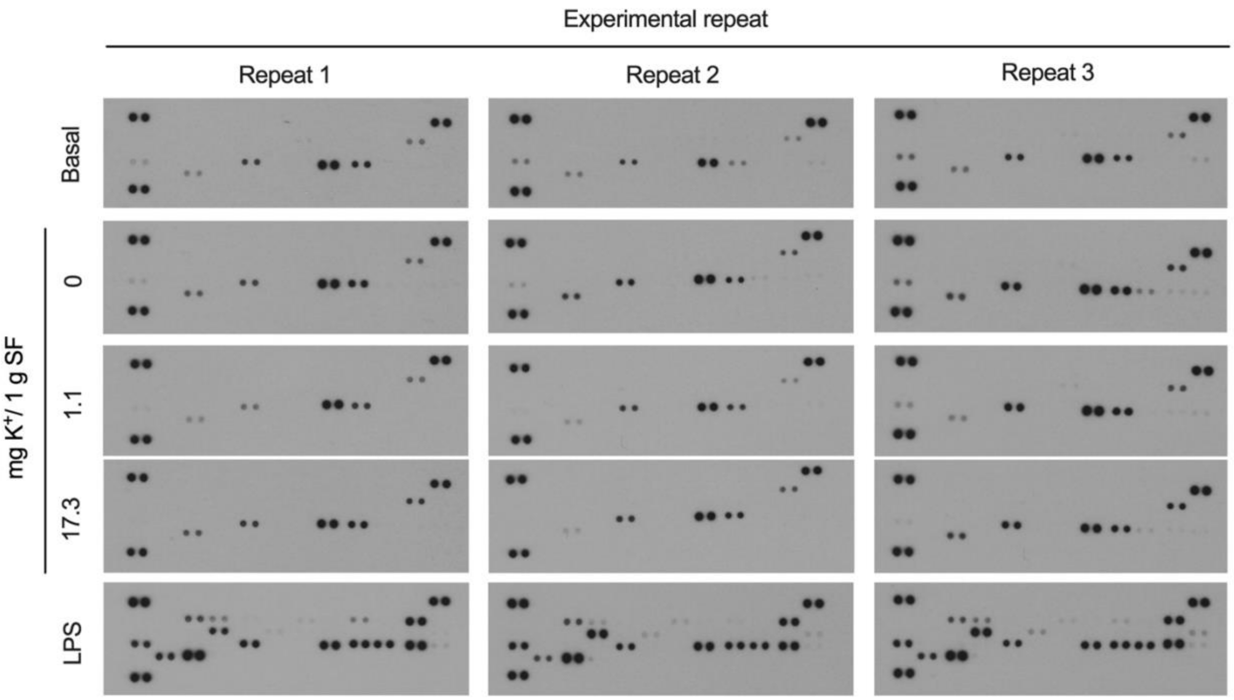

Figure S4. Inflammatory cytokine levels were measured in RAW 264.7 murine macrophages in response to silk nanoparticles. Cytokine expression data were collected from three independent experimental repeats. Macrophages were treated with either complete media or media containing 200 ng/mL lipopolysaccharide, which served as the basal and positive controls, respectively. This experiment was carried out alongside our calcium silk nanoparticle,[26] with the same set of controls used for both. Cytokines and chemokines were processed following the manufacturer's guidelines (n = 3). Abbreviations: aqueous silk fibroin (SF); lipopolysaccharide (LPS).
